# Localization and classification of space objects using EfficientDet detector for space situational awareness

**DOI:** 10.1038/s41598-022-25859-y

**Published:** 2022-12-19

**Authors:** Nouar AlDahoul, Hezerul Abdul Karim, Angelo De Castro, Myles Joshua Toledo Tan

**Affiliations:** 1 Faculty of Engineering, Multimedia University, Cyberjaya, Malaysia; 2 College of Engineering and Technology, University of St. La Salle, 6100 Bacolod, Philippines; 3 New York University Abu Dhabi, Abu Dhabi, UAE

**Keywords:** Computational science, Computer science, Information technology

## Abstract

Space situational awareness (SSA) systems play a significant role in space navigation missions. One of the most essential tasks of this system is to recognize space objects such as spacecrafts and debris for various purposes including active debris removal, on-orbit servicing, and satellite formation. The complexity of object recognition in space is due to several sensing conditions, including the variety of object sizes with high contrast, low signal-to-noise ratio, noisy backgrounds, and several orbital scenarios. Existing methods have targeted the classification of images containing space objects with complex backgrounds using various convolutional neural networks. These methods sometimes lose attention on the objects in these images, which leads to misclassification and low accuracy. This paper proposes a decision fusion method that involves training an EfficientDet model with an EfficientNet-v2 backbone to detect space objects. Furthermore, the detected objects were augmented by blurring and by adding noise, and were then passed into the EfficientNet-B4 model for training. The decisions from both models were fused to find the final category among 11 categories. The experiments were conducted by utilizing a recently developed space object dataset (SPARK) generated from realistic space simulation environments. The dataset consists of 11 categories of objects with 150,000 RGB images and 150,000 depth images. The proposed object detection solution yielded superior performance and its feasibility for use in real-world SSA systems was demonstrated. Results show significant improvement in accuracy (94%), and performance metric (1.9223%) for object classification and in mean precision (78.45%) and mean recall (92.00%) for object detection.

## Introduction

Over the past few decades, operations carried out by space organizations, such as the National Aeronautics and Space Administration (NASA) and the European Space Agency (ESA), have resulted in enormous amounts of space debris being sent into orbit around the planet. Space agencies' operations are mostly focused on the navigation of the solar system, weather monitoring on Earth, and space launch campaigns, to name a few examples. The Space Situational Awareness Initiative (SSA) aims to equip Europe and its residents with full and reliable data on objects circling the Earth, and on the dangers originating in space. By fulfilling its objectives, the SSA program will allow Europe to independently identify, anticipate, and evaluate the dangers to people and property posed by various perils that could happen in the solar system^1^.

In recent years, several works have been presented to study the possible benefits of artificial intelligence (AI), particularly deep learning (DL), on improving accuracy of the classification and detection of objects in photographs taken for space operations^2–7^. The accessibility and quality of information needed to train deep learning systems have a significant impact on their efficiency, as has been demonstrated in various studies^8–10^. Data, on the other hand, are extremely rare and expensive to gather in the space domain. In addition, various research has been conducted utilizing vision-based sensors, and to perform unsupervised near-earth missions in space with refractory targets, and durable and fast onboard posture stabilization techniques that are necessary on the spacecraft^11–13^. As a result, various image-based studies suggest the use of motion sensors such as Light Detection and Ranging (LiDAR) to achieve this goal^14–16^. To utilize neural network models successfully, a lot of data are needed. Monitoring the encircling components around the spacecraft is challenging since they vary in size, shape, and composition. Obtaining data from these spacecrafts is also a costly task. For this reason, research teams have begun to examine satellite data collection. The SPARK dataset offers a realistic representation of the earth and of the objects in and around it^14,17,18^.

Various object recognition algorithms have been published over the last decade, and it is fascinating to explore the suitability of these techniques to space data, as well as to find ways for improving their efficiency in the space domain. Unsupervised object detection is performed by convolutional neural networks (CNNs), which eliminate the need for features to be generated and obtained individually^19^. Deep learning-based techniques use CNNs to do this task.

## Related works

The region-based convolutional neural network (R-CNN) family of object identification algorithms includes a variety of widely used object detection techniques^20^. Premised on the region proposal architecture, which is an extended version of the linear regression technique and is also used by Faster R-CNN^21^, these frameworks have been designed and implemented. It is believed that this decoder will find objects in portions of an image where the algorithm anticipates they may be present^22^. As technology progresses, algorithms, likewise, become more precise but also become more computationally expensive. Mask R-CNN, designed by developers at Facebook, is one of the most recent algorithms that serves as a useful initial point for object detection models on the client side of the network^23^. On the other hand, single shot detectors (SSDs) are designed to depend on a fixed number of regions instead of a subnetwork to suggest regions. Upon overlaying an input image, a grid of reference points is created, and at each point, boxes of various shapes and sizes are used to define the areas^24^. There are also a variety of versions available that are part of the single shot detector network. The encoders used in each model, as well as the precise layout of predefined points, are the primary differences between them. The MobileNet + SSD models include a MobileNet-based encoder^25^, while the YOLO model includes a convolutional architecture that is proprietary to it. The YOLO concept takes a completely distinct approach. It uses a single neural network to process the entire picture. For each region, this network separates the picture into regions, from which it anticipates the bounding boxes and probabilities^26–28^. Thus, the weighting of these bounding boxes is determined by the projected probability. For this reason, SSDs are excellent alternatives for models that could be used in mobile or embedded systems. Furthermore, in a recent publication, the Google Brain team described their EfficientDet architecture for object detection, intending to design selections into a scalable structure that can be used for a variety of diverse object detection applications^29^. On standard datasets, the study suggests that EfficientDet performs better simulations of equal size.

To reduce the possibility of collisions occurring in space, the process of target recognition ought to be carried out automatically^30^. The most important component in SSA for analyzing visual data and developing data-driven AI solutions is the vision-based sensor^13–15,31^, which can take the form of a camera. On one hand, past research papers have presented a variety of technologies to detect and manage active and inactive satellites, while on the other hand, several strategies have been suggested to eliminate debris from space. In addition, LiDAR sensors have been utilized for the removal of debris, the recognition of targets, and the estimation of poses^13–16^. It was discovered that there are ways for estimating the pose of a 3D spacecraft by comparing the wireframe of the target with a 2D image. These approaches make use of a matching process that compares visual elements taken from the image and the wireframe^5^. In order to find the pose, the Perspective-n-Point (PnP) argument needed to be solved^5^. In order to extract the edge characteristics, traditional computer vision methods such as Sobel and Canny detectors were utilized^32,33^. On the other hand, conventional machine learning algorithms were taken into consideration for the task of posture estimation utilizing principal component analysis (PCA)^34^. After applying PCA to a spacecraft image in question, the results were compared with the ground truth postures contained inside the dataset for the objective of matching. Detecting objects, determining their bounding boxes, and classifying images are some of the most important challenges in computer vision. Object detection and image classification are used to accomplish these goals. Deep learning techniques, which use automatic feature learning and extraction, have been shown to generate superior outcomes over other computer vision methods. As a consequence, deep learning algorithms have been implemented in space applications with the goal of recognizing spacecraft and debris for a variety of reasons. One of the deep learning models that were utilized to estimate the posture of the spacecraft was a pre-trained convolutional neural network^6,35^, such as GoogleNet^7,36^. Similarly, VGG^37,38^ has been trained and evaluated on a synthetic dataset in order to identify the translation and rotation of a space object relative to a camera. In addition, ResNet was presented for the purpose of estimating the pose of an uncooperative spacecraft without the use of any 3D input and for predicting the bounding box of space objects^5,10^. The quantity of information that is input into the deep learning model is directly related to both the effectiveness of the method and its capacity for generalization. In order to achieve the desired level of efficiency in comparison to more conventional machine learning strategies, the size of the data set used must be substantial. The expense of acquiring data from spacecrafts is quite high. As a result of this, many different synthetic datasets have been presented in research works for the purpose of 6D pose estimation. Two examples of these are the Unreal Rendered Spacecraft On-Orbit (URSO) dataset^6^ and the Spacecraft posture estimation dataset (SPEED)^39,40^. The fact that the nearby spacecraft or objects are of varying sizes makes object surveillance a difficult and complicated process. This is in addition to the high cost of acquiring space data. Researchers have taken into consideration the technique of data gathering to collect images of space objects such as spacecraft and debris in order to solve the issues that were previously identified. Because of this, they created a high-resolution synthetic spacecraft dataset by using the environment simulator that comes with the Unity3D gaming engine^41^. In order to provide an adequately labelled space dataset, a new SPARK dataset was put together and was designed exclusively for the classification of space objects^14,18^. The SPARK dataset portrays a genuine earth and other objects located in its immediate vicinity. Both ResNet^10^ and EfficientNet^42^ were presented as examples of pre-trained CNNs that made use of the SPARK dataset and a number of different examples^14^. The three possible outcomes are as follows: (1) initializing the models with random data and beginning the training process from scratch; (2) feature extraction by freezing the backbone of the network and only training the classifiers in the top layers of the network; and (3) making use of the pre-trained weights and then fine-tuning on the entire model, including the backbone and the classifier. It was discovered that the algorithms that were trained on both RGB and depth pictures performed significantly better than single models^14^.

AlDahoul et al.^43^ have proposed a multi-modal learning method with SPARK dataset. They formulated the problem as an image classification problem to identify the space object category directly from the whole image applied to the CNN. The features were extracted from RGB images of spacecraft and debris, utilizing numerous convolutional neural networks such as DenseNet, ResNet, and EfficientNet. They also explored vision transformer for same purpose. For depth images classification, the End-to-End CNN was demonstrated. They have found that combining RGB based vision transformer and depth-based End-to-End CNN produced better performance in terms of accuracy and F1 score.

On the other hand, localization of space objects before classification was proposed to focus attention on regions of space objects and to ignore other irrelevant objects in the background^2^. Their detection algorithm did not use traditional object detectors, such as YOLO and faster R-CNN, which require annotation with bounding boxes for objects in each image. They implemented a simple detection algorithm on depth images in a few steps: (1) smoothing images using a Gaussian filter; (2) up-sampling images twice to produce the depth images that have the same size as the RGB image; and (3) converting images to black-and-white by thresholding and inverting them. After obtaining cropped images that have only space objects in RGB and depth versions, a decision fusion approach was applied.

This study demonstrates the utilization of an object detection method using an EfficientDet model that has been found to outperform other object detectors for various applications^44^. The first objective is to enhance classification performance by focusing attention on regions of space objects and ignoring other irrelevant objects in the background. This contributes to the improvement of accuracy and performance metrics when compared with existing solutions. Moreover, localization of space objects in the image by predicting four coordinates of the object is the second significant objective that helps SSA systems in space navigation missions.

The study presented in this paper aims to attract the research community by highlighting an interesting new challenge that enriches the body of knowledge by proposing the following:

1. A space object detection model that localizes debris and spacecraft objects in RGB-based space images and that classifies them into eleven classes;
2. A multi-modal learning approach for spacecraft classification that uses only RGB images to combine decisions from efficientNet-v2 and EfficientNet-B4;
3. An evaluation of metrics and comparison with methods utilized for the same purpose of space object classification.
4. An ablation study to validate significant improvements in classification accuracy by using multi-modal learning, which yield the final decision by combining decisions from efficientNet-v2 and EfficientNet-B4 CNNs.

The organization of this paper is as follows: The description of the SPARK space imagery dataset was done in section “Materials and methods”. Furthermore, the approaches to object detection and multi-modal learning were also demonstrated in this section. Section “Results and discussion” discusses the experiments conducted in this study and analyses the results by comparing the proposed method with the existing solutions. Finally, in section “Conclusion and future work”, we summarized the outcome of this work to give the readers a glimpse into potential improvements in the future.

## Materials and methods

The description of the dataset used in this work is presented in this section to highlight the challenging contents available in this dataset of space images. Furthermore, the object detection method of EfficientDet is demonstrated to shed light on its superiority over state-of-the-art object detectors. Additionally, a decision fusion approach is discussed to study the efficiency of fusing decisions from two models.

### Datasets overview

This research makes use of a unique space dataset to address the ICIP 2021 issue of Spacecraft Recognition leveraging Knowledge of the Space Environment (SPARK)^14,17,18^. The collection contains 150,000 RGB pictures and 150,000 depth photos. This dataset was utilized to categorize 11 different types of objects, comprising 10 satellite systems.

**Figure 1:**
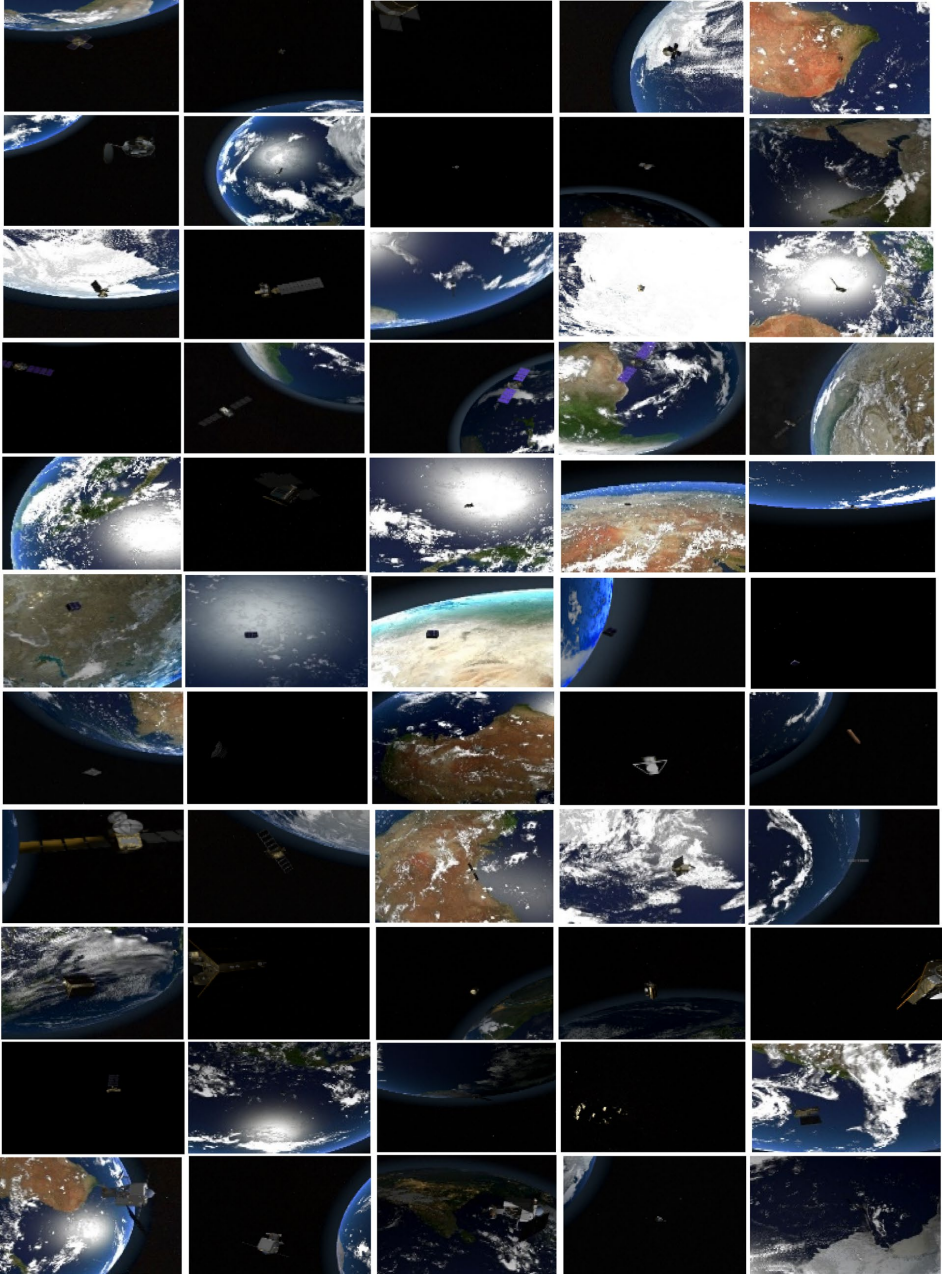
Few samples of RGB images with various object sizes and backgrounds from the spark dataset^14,18^ including AcrimSat, Aquarius, Aura, Calipso, Cloudsat, CubeSat, Debris, Jason, Sentinel-6, Terra, and TRMM in the rows 1, 2, 3, 4, 5, 6, 7, 8, 9, 10, and 11 respectively^14,17,18^.

Figure 1 shows few samples of RGB images from the SPARK dataset^14,17,18^. These samples summarize the challenges present in this dataset including random locations of objects, illuminated stars and increased contrast, a variety of orbital settings, various positions and orientations of space objects in the background, the earth having oceans and clouds in the background, a substantial noise level, and various object sizes.

### EfficientNet algorithm

To begin training an object identification model, images are converted into unique features that are applied to the inputs of neural networks. By utilizing CNNs to extract trainable characteristics from images, significant development has been achieved in the discipline of computer vision^19^. CNNs combine and pool picture information at several granularities, providing the model with a variety of potential configurations to focus on while learning the image identification tasks at hand.

EfficientNet is the foundation of the EfficientDet framework. EfficientNet started to investigate how CNN designs to scale^42^. There are various techniques, but it turns out that users can augment a CNN with additional parameters. Users may increase the width of each layer, the depth of the layers, or the resolution of the photos entered, or users can do a variety of these things. EfficientNet intended to develop a method for scaling CNN structures automatically^42^. The purpose of their work is to improve downstream efficiency with available free-range over depth, breadth, and resolution while remaining within the limits of target memory and FLOPs^29^.

It is the goal of feature fusion to merge samples of a particular image that are captured at various resolutions. Traditionally, the fusion employs the final several feature layers from the CNN, although the specific neural network used may differ.

### EfficientDet model

Feature pyramid network (FPN) is a standard method for fusing features with a top-down direction^45^. The Path Aggregation networks (PANet) enables reverse and forward flows of feature fusion from lower to higher resolution^46^. Consequently, NAS-FPN is a feature fusion approach developed via neural architecture search (NAS)^47^. Finally, the EfficientDet model stacks these BiFPN blocks. The model scaling process alters the number of blocks.

A scaling issue was created to dynamically resize the backbone, Weighted Bi-directional FPN (BiFPN), class/box, and input image quality. The network structure scales automatically with EfficientNet-B0 to EfficientNet-B6. Thus, the amount of BiFPN stacks affects the network depth and breadth^29^. The EfficientDet framework is validated on 100,000 photos from the COCO (Common Objects in Context) dataset. Success in this area implies success in smaller particular activities. In many cases, EfficientDet outperforms other object detection methods^29^.

The authors of EfficientNet constructed the foundation model by employing a multi-objective neural network system that maximizes both efficiency and FLOP. Also, the equation they utilized is inspired by MnasNET, as seen in the Eq. (1)^48^.

1 $$\begin{array}{*{20}c} {{\text{maximize}}} \\ m \\ \end{array} ACC\left( m \right) \cdot \left[ {\frac{FLOPS\left( m \right)}{T}} \right]^{w}$$

$ACC(m)$ and $FLOPS(m)$ is expressed as the accuracy and the FLOPS of the algorithm $m$, $T$ is the FLOPS’ target, and $w =- 0.07$ is a hyperparameter that regulates the exchange among accuracy and FLOPS (floating point operations per second). Their investigation resulted in the discovery of an efficient network, which they termed EfficientNet-B0. The EfficientNet appears to be a solid foundation upon which to develop. It shows how easily scales with model performance and outperforms other CNN backbones, as demonstrated by its superior performance.

It is recommended that the BiFPN function as the feature network, where it accepts levels three to seven elements (P3,P4,P5,P6,P7) from the backbone network (EfficientNet) and implements simultaneous feature fusion top-down and bottom-up continuously^29^.

2 $$W_{BiFPN} = 64 \cdot \left( {1.35^{\phi } } \right),D_{BiFPN} = 3 + \phi$$

where φ = 0 for EfficientDet-D 0,1,2, … , 7 for EfficientDet-D7.

Because its level of BiFPN must be converted to tiny integers, the authors exponentially extend the width of BiFPN (#channels), as was conducted in EfficientNets, but steadily improves the depth (#layers) and it is expressed using the formula in Eq. (2). The width is maintained at the exact level of the BiFPN. However, the depth (number of layers) is raised continuously and expressed in the following equation^29^:

3 $${D}_{box}={D}_{class}=3+\left[\frac{\phi }{3}\right]$$

Considering that BiFPN employs feature levels three to seven, the input resolution has to be divisible by ${2}^{7}=128$, which means that it linearly enhances resolutions applying the following formula^29^:

4 $${R}_{input}=512+(\phi )(128)$$

In general, an improved compound scaling approach for object recognition was presented, wherein it makes use of a simple compound coefficient, $\phi$, to simultaneously scale-up all features of the backbone structure, featured network, class/box network, and the input image resolution.

The EfficientDet Architecture is built on the backbone network EfficientNet^42^. Both feature network BiFPN and class/box net layers are reiterated numerous times to account for resource restrictions of varying magnitude^29^.

### The proposed solution

This section discusses the proposed system of decision fusion that combines the EfficientDet model with an EfficientNet-v2 backbone to localize and classify space objects and EfficientNet-B4 model to classify the cropped images that contain space objects.

**Figure 2:**
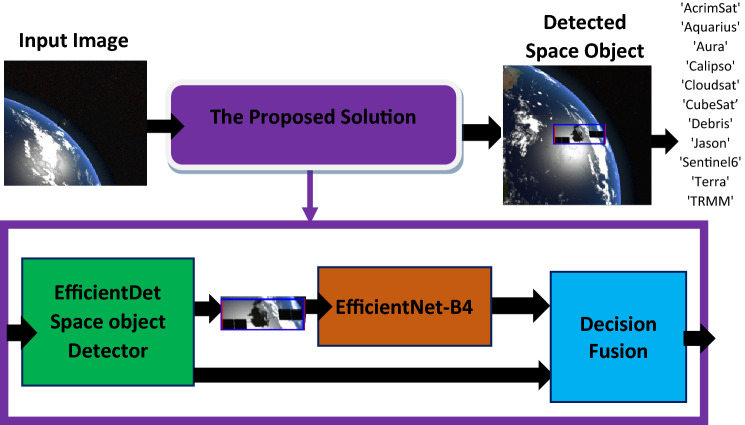
The block diagram of the proposed Solution. The images were taken from^14,17,18^.

First, the experiments were conducted to train the EfficientDet object detector. In this detector, we selected EfficientNet-v2 as a backbone because it has shown superior balance between accuracy and speed in the literature. The hyperparameters were selected carefully to guarantee high performance of detection. After detector training, the evaluation metrics showed high performance in localization stage. Additionally, the detector was able to classify most of space objects with high accuracy. However, the detector was not able to classify specific category “CloudSat” of spacecraft which led to accuracy drop off. After investigation we found that testing samples of “CloudSat” category have noisy and blurred images which were not available in the training set. To address the previously mentioned problem, the cropped images that have the detected objects were augmented by adding blurring and noise. After that, these new set of training samples that contain cropped images of all categories with blurred and noisy versions of “CloudSat” samples were passed to EfficientNet-B4 CNN for training. Finally, the decisions from both models (EfficientDet and EfficientNet-B4) were fused to find the final category among eleven categories. The fusion was done by checking the prediction outcome of EfficientNet-B4 if it has “CloudSat” category, this would be the final decision. Otherwise, the final decision would be the prediction outcome of EfficientDet. Figure 2 shows the block diagram of the proposed Solution.

## Results and discussion

### Experimental setup

**Figure 3:**
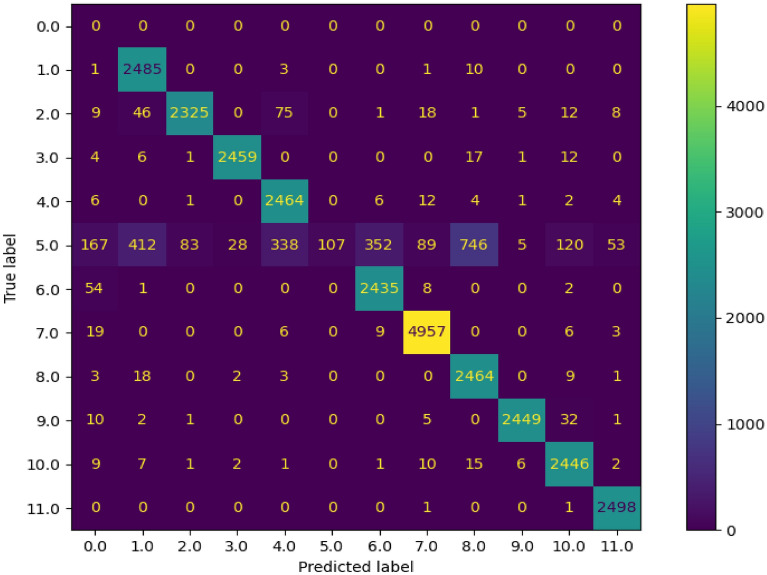
Confusion Matrix of EfficientDet for 11 categories of the SPARK dataset.

**Figure 4:**
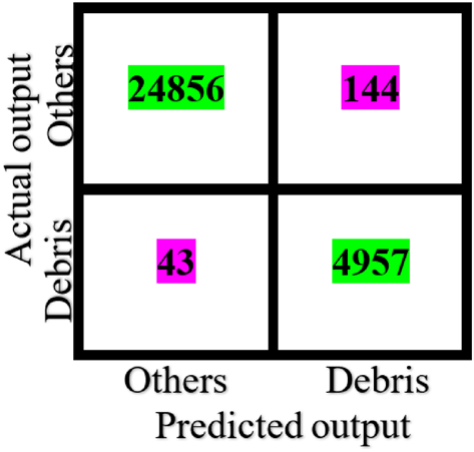
Confusion Matrix of EfficientDet for Debris/Satellite classification of SPARK dataset.

**Figure 5:**
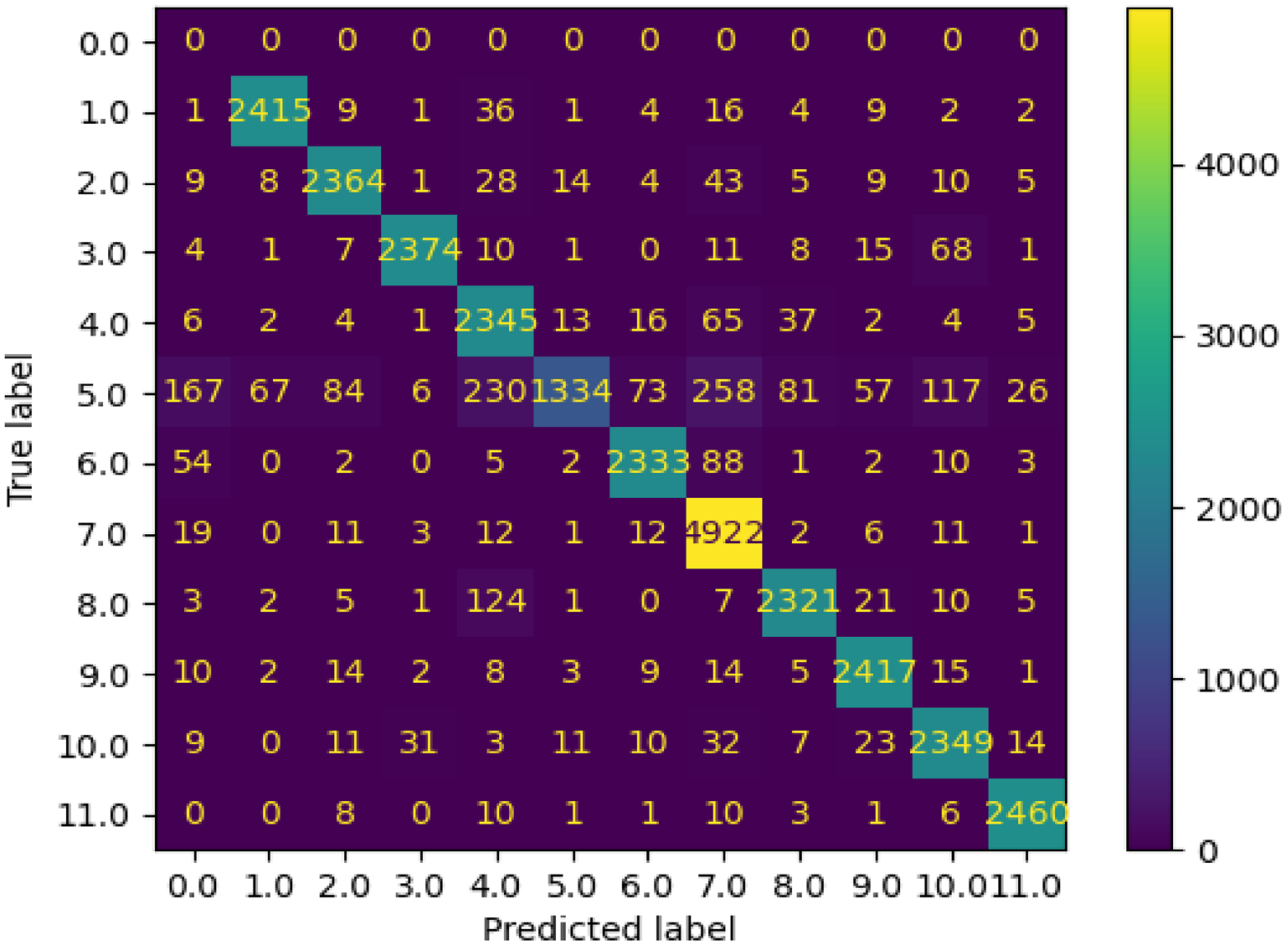
Confusion Matrix of EfficientNet-B4 with cropped images of SPARK dataset for 11 categories.

**Figure 6:**
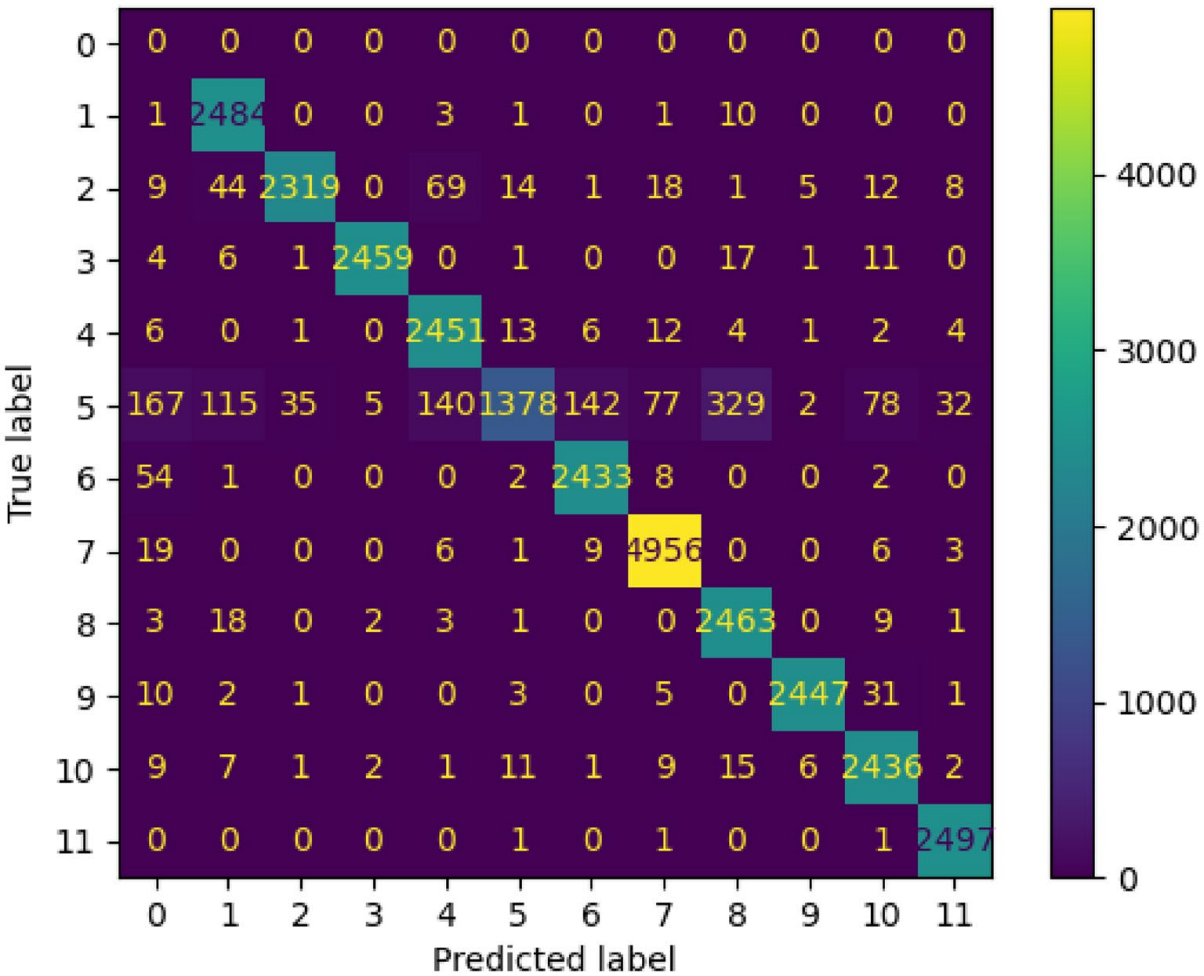
Confusion Matrix of the proposed solution with SPARK dataset for 11 categories.

**Figure 7:**
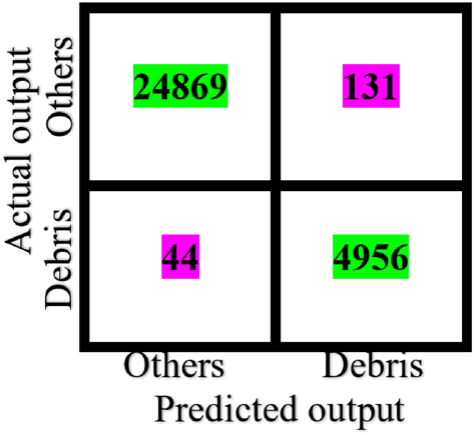
Confusion Matrix of the proposed solution with SPARK dataset for debris/satellite classification.

For the SPARK dataset, only training and validation sets were provided with labels. Therefore, we divided the training set into two sets: 80% (72,000 images) for training, and 20% for validation (18,000 images). On the other hand, validation dataset that includes 30,000 images was used for testing. The results shown in Tables 1, 2, and 3 and in Figs. 3, 4, 5, 6, and 7 belong to the results of the testing dataset. The experiments conducted for this research work were done using the PyTorch and TensorFlow frameworks on an NVIDIA Tesla V100 GPU.

**Table 1:** Classification accuracy, precision, recall, and F1-score, of the EfficientDet with the SPARK dataset.

Category	Accuracy %	Precision %	Recall %	F1-score %
AcrimSat	99.4	83	99	91
Aquarius	93.0	96	93	95
Aura	98.36	99	98	99
Calipso	98.56	85	99	91
Cloudsat	04.28	100	04	08
CubeSat	97.4	87	97	92
Debris	99.14	97	99	98
Jason	98.56	76	99	86
Sentinel-6	97.96	99	98	99
Terra	97.84	93	98	95
TRMM	99.92	97	100	99
Average	89.5	84	82	79

**Table 2:** Classification accuracy, precision, recall, and F1-score, of the EfficientNet-B4 with cropped images of SPARK dataset.

Category	Accuracy %	Precision %	Recall %	F1-score %
AcrimSat	96.6	97	97	97
Aquarius	94.56	94	95	94
Aura	94.96	98	95	97
Calipso	93.8	83	94	88
Cloudsat	53.36	97	53	69
CubeSat	93.32	95	93	94
Debris	98.44	90	98	94
Jason	92.84	94	93	93
Sentinel-6	96.68	94	97	95
Terra	93.96	90	94	92
TRMM	98.4	98	98	98
Average	91.54	86	84	84

**Table 3:** Classification accuracy, precision, recall, and F1-score, of the proposed solution with SPARK dataset.

Category	Accuracy %	Precision %	Recall %	F1-score %
AcrimSat	99.36	93	99	96
Aquarius	92.76	98	93	95
Aura	98.36	100	98	99
Calipso	98.04	92	98	95
Cloudsat	55.12	97	55	70
CubeSat	97.32	94	97	96
Debris	99.12	97	99	98
Jason	98.52	87	99	92
Sentinel-6	97.88	99	98	99
Terra	97.44	94	97	96
TRMM	99.88	98	100	99
Average	93.98	87	86	86

The first bag of experiments was carried out for space object detection. The space images were resized to 512 × 512 before being applied to the input of the EfficientDet detector. Additionally, the images were normalized using the mean and standard deviation of the ImageNet dataset. The number of epochs was set to 10. The batch size was 4. The learning rate was 0.0002.

The second bag of experiments was carried out for space object classification using the EfficientNett-B4 CNN. The cropped images that resulted from the detector were resized to 224 × 224 before being applied to the input of the EfficientNet-B4 CNN. The layers of the base model were frozen with ImageNet weights. The last twenty layers were trained with space images. Additionally, the top layers were replaced by the following layers:

1. GlobalAveragePooling2D layer
2. BatchNormalization layer
3. Dropout layer with 0.2
4. Dense layer with 11 nodes

The hyperparameters are as follows:

1. learning rate of 0.0001
2. Optimizer of Adam
3. Loss function of Categorical Cross-entropy
4. Batch size of 64
5. Number of epochs of 12

### Classification evaluation metrics

To evaluate the classification performance, several metrics, namely accuracy, precision, recall, F1 score, F2 score, and Perf were utilized. This section defines the performance metrics as follows:

1. Accuracy is a measure that calculates number of samples predicted correctly over all available samples.
  5 $$Accuracy =\frac{TP+TN}{TP+TN+FP+FN}$$
2. Recall (Sensitivity) is a measure that calculates the proportion of actual positives that are identified correctly
  6 $$Recall = \frac{TP}{TP+FN}$$
3. Precision (positive predictive value) is a measure that calculates the proportion of positive identifications that are correct
  7 $$Precision=\frac{TP}{TP+FP}$$
  where TP: True Positive, TN: True Negative, FP: False Positive, FN: False Negative.
4. F1 score is a metric that summarizes recall and precision into a single term.
  8 $$F1 score = \frac{2 \times precision \times recall}{precision+recall}$$
5. F2 score is a weighted harmonic mean of precision and recall. It was used to avoid misclassification of debris as satellites.
  9 $$F2 score (debris) = \frac{5 \times precision \times recall}{4 \times precision+recall}$$
6. Perf metric is a metric that is given as follows:
  10 $${\text{Perf }} = {\text{ F2 score }}\left( {{\text{debris}}} \right) \, + {\text{ Accuracy }}\left( {{\text{Satellites}}} \right)$$

### Detection evaluation metrics

To evaluate the detection performance, several metrics such as Intersection Over Union (IOU), mean recall, and mean precision were utilized. This section describes the performance metrics as follows:

1. Intersection Over Union
  B: area covered by ground-truth bounding boxes.
  B′: area covered by predicted bounding boxes.
  IOU is an object detection metric used to measure the overlap between the actual bounding box and th predicted bounding box. A greater IoU value means a greater overlap and better detection performance. It is calculated by dividing the area of the intersection of the two boxes over the area of the union of the two boxes.
  11 $${\text{IoU }}({\text{B}},{{{\rm B}^{\prime}}}) \, = \frac{{B \cap B^{\prime} }}{{B \cup B^{\prime} }}$$
2. Recall
  The recall in a detection task is related to the inability of an algorithm to detect objects present in the image by producing false negatives. We calculated the average recall of all classes at each IoU threshold and then calculated the mean as shown in Table 6. Additionally, we plotted an Recall vs. IoU curve with IoU thresholds on the x-axis and recall on the y-axis. This plot illustrates the recall for each class vs. IOU thresholds ∈ [0.5, 9.5] as shown in Fig. 10.
3. Precision
  The precision in a detection task is related to incorrect detection of irrelevant things in the background as an object. It can be determined by utilizing the IoU threshold. If the IoU is smaller than the threshold, it is classified as a false positive. On the other hand, if an IoU is bigger than the threshold, it is classified as a true positive. We calculated the average precision of all classes at each IoU threshold and then calculated the mean as shown in Table 6. Additionally, we plotted Precision vs. IoU curve with IoU thresholds on the x-axis and precision on the y-axis. This plot illustrates the precision for each class vs. IOU thresholds ∈ [0.5, 9.5] as shown in Fig. 11. A model is considered as a good model if it has high precision and high recall.
4. Confidence Score
  This score reflects how accurate the bounding box is and how likely there is to be an object. If no object exists, the confidence score is zero.

### Experimental results

In this section, we present the results of experiments conducted to detect (localize and classify) the space objects in images of the SPARK dataset. Additionally, we evaluate the performance of the proposed solution and compare it with various baseline methods that were proposed recently in the literature. We divided the performance evaluation into two parts: classification performance evaluation and detection performance evaluation.

#### Classification performance evaluation

To measure classification performance, the accuracy, precision, recall, and F1 score were calculated for each class of eleven classes, and then averages were determined. The results of accuracy, precision, recall, and F1 score are shown in Tables 1, 2, and 3 for the following three methods:

1. EfficientDet with EfficientNet-v2 backbone.
2. EfficientNet-B4 CNN used with cropped images.
3. decision fusion method.

The average accuracy, precision, recall, and F1 score of EfficientDet with EfficientNet-v2 backbone were 89.5%, 84%, 82%, 79% respectively as shown in Table 1.

Figure 3 shows the confusion matrix of the of EfficientDet with EfficientNet-v2 backbone. The high values of the elements in the main diagonal are clear. In this confusion matrix, the labels are numbered from 1 to 11 to represent the following categories: AcrimSat, Aquarius, Aura, Calipso, CloudSat, CubeSat, Debris, Jason, Sentinel-6, Terra, and TRMM, respectively. The samples with label 0 refer to the mis-detected samples. In other words, the model mis-detected 1 sample from first category, 9 samples from second category, and so on. 167 was the largest number of mis-detected samples from the “CloudSat” category.

The samples with label 5 which represent the “CloudSat” category were misclassified as labels 1, 4, 6, and 8. Only 107 out of 2500 samples were classified correctly. The reason was that images in the “Cloudsat” category during the initial testing set were noisy and blurry and were different from the images in the training set.

The confusion matrix of the binary debris/satellite classification task that used the EfficientDet model is shown in Fig. 4. The matrix is evidence of the high capability of the classifier to identify debris out from other categories.

The average accuracy, precision, recall, and F1 score of EfficientNet-B4 with cropped images were 91.54%, 86%, 84%, 84%, respectively as shown in Table 2.

Figure 5 shows the confusion matrix of the EfficientNet-B4 model with cropped images. The high values of the elements in the main diagonal are clear. The number of samples with label 5, which represents “CloudSat” category, has been increased remarkably compared to the previous EfficientDet model. In other words, 1334 out of 2500 samples were classified correctly. The reason was that we augmented images with “Cloudsat” category in the training set by adding blurring and noise and then passed the set into the EfficientNet-B4 model for training.

The average accuracy, precision, recall, and F1 score of the proposed solution of decision fusion were 93.98%, 87%, 86%, 86%, respectively as shown in Table 3.

Figure 6 shows the confusion matrix of the of the proposed solution of decision fusion. The high values of the elements in the main diagonal are evident. The number of samples with label 5 which represents the “CloudSat” category has been increased compared to the previous EfficientNet-B4 CNN. In other words, 1378 out of 2500 samples were classified correctly. The reason was that we combined the decisions from two previously mentioned models that include EfficientDet with an EfficientNet-v2 backbone and EfficientNet-B4 CNN.

The confusion matrix of binary debris/satellite classification of the proposed solution of decision fusion is shown in Fig. 7. The matrix shows the high capability of the classifier to identify debris out from other categories.

#### Ablation study

In this section, an ablation study is described to validate the significance of decision fusion that made the final decision by combining decisions from EfficientDet with an EfficientNet-v2 backbone and EfficientNet-B4 CNN. The proposed solution was compared with the baseline methods in terms of accuracy, F2-score, and Perf metric as shown in Table 4. The accuracy here is related to only 10 categories of satellites and ignore the “debris” category that the F2 score focuses on.

**Table 4:** Classification accuracy, F2-score, and Perf metric of the proposed solution with SPARK dataset.

Solution	Accuracy %	F2-score %	Perf metric
Multimodal CNNs (baseline)^2^	0.8677	0.9539	1.8216
EfficientDet-with EfficientNet v2 backbone (proposed)	0.8853	0.9874	1.8727
The proposed solution	0.9346	0.9877	1.9223

It was found that decision fusion was able to make significant improvements in classification over EfficientDet by increasing the accuracy from 88.53 to 93.46% and the performance metric from 1.8727 to 1.9223.

The proposed solution for decision fusion that combines decisions from EfficientDet with an EfficientNet-v2 backbone and EfficientNet-B4 CNN was compared with the baseline method in terms of accuracy, F2-score, and Perf metric as shown in Table 4. The baseline method is the multimodal CNNs^2^ that includes a pre-trained ResNet50 CNN connected to a support vector machine (SVM) classifier for classification of RGB images and an end-to-end CNN for classification of depth images. It was found that the proposed solution was able to make significant improvements in classification by increasing accuracy from 86.77 to 93.46%, F2 score from 95.39 to 98.77%, and performance metric from 1.8216 to 1.9223.

In Table 5, the proposed solution was also compared with the baseline methods in the literature in terms of accuracy. The accuracy here refers to the average accuracy of all 11 categories including satellites and debris. In^2^, the authors used ResNet50 CNN + SVM with cropped RGB images only, just as our proposed method does, and yielded 85% accuracy. Then, they proposed multimodal CNNs using both RGB and depth images after detection and cropping to increase accuracy from 85 to 89% as shown in Table 5. Additionally, AlDahoul et al.^43^ proposed various methods to recognize spacecrafts as shown in Table 5. Some methods utilized only RGB images, just as ours does. A vision transformer was utilized with whole RGB images without detection and yielded 81% accuracy. On the other hand, some methods in^43^ used both RGB images and depth images to improve the recognition accuracy. Both EfficientNetB7-End2End CNN and Vision Transformer-End2End CNN have accuracies of 85% using also whole images without detection.

**Table 5:** Comparison between the proposed solution and state-of-the-art methods in terms of accuracy using SPARK dataset.

Method	Accuracy %
Multimodal CNNs (baseline)^2^	89
ResNet50 CNN + SVM—RGB only (baseline)^2^	85
Vision Transformer—RGB only (baseline)^43^	81
Multi-modal (EfficientNetB7—End2End CNN) (baseline)^43^	85
Multi-modal (Vision Transformer- End2End CNN) (baseline)^43^	85
EfficientDet-with EfficientNet v2 backbbone—RGB only (proposed)	**89.5**
EfficientNet-B4 with cropped images- RGB only (proposed)	**91.5**
The proposed solution	**94**

Significant values are in [bold].

It is obvious in Table 5 that our proposed methods can outperform other methods in terms of accuracy, utilizing only RGB images to produce 89.5% with EfficientDet alone, 91.5% with EfficientNet-B4 alone, and 94% with decision fusion that combines decisions from EfficientDet with an EfficientNet-v2 backbone and EfficientNet-B4 CNN.

#### Detection performance evaluation

**Figure 8:**
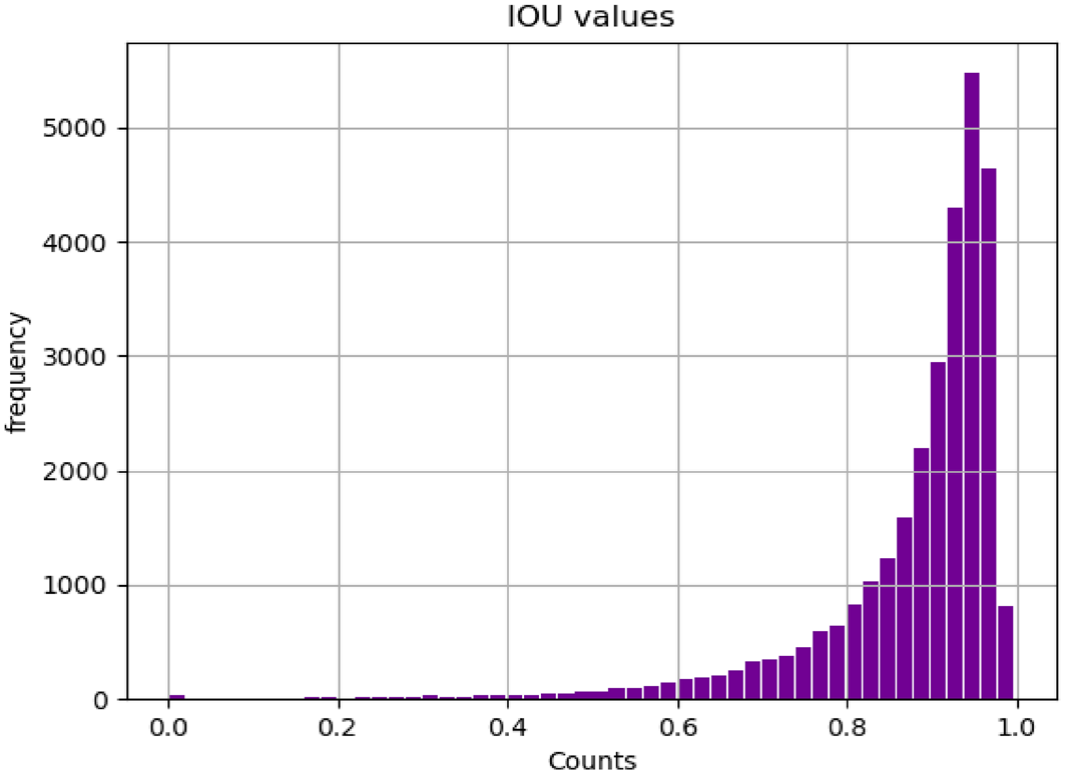
Distribution of IOU in EfficientDet model.

**Figure 9:**
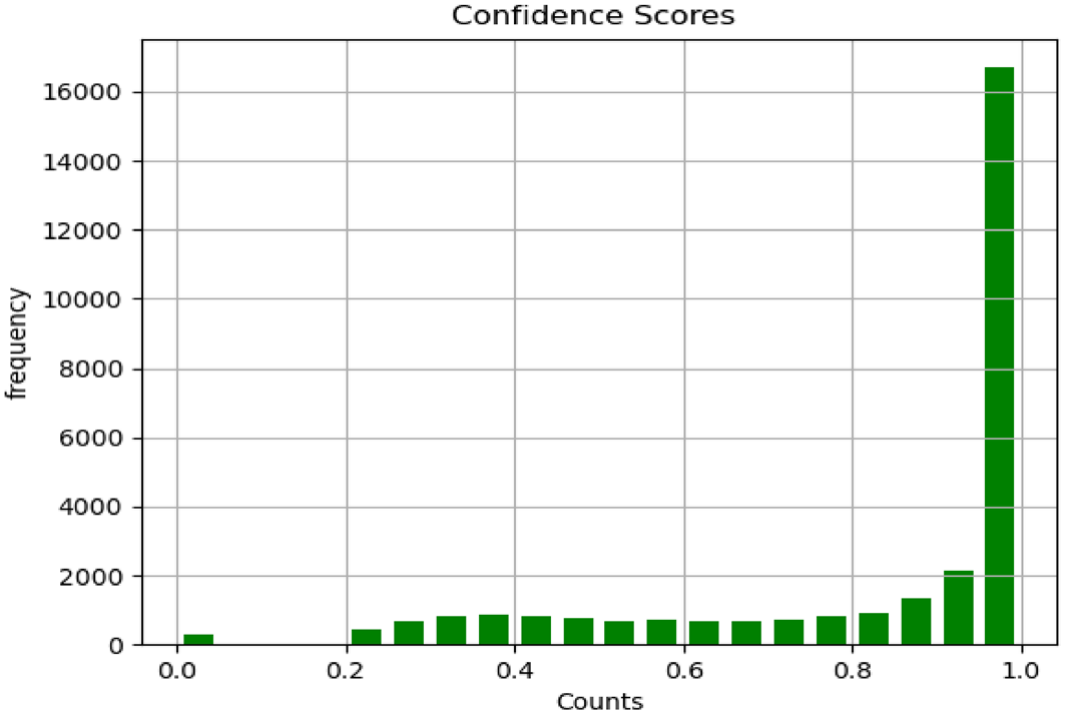
Distribution of Confidence scores in EfficientDet model.

To evaluate the detection model, a distribution of IoUs between ground truth bounding boxes and predicted bounding boxes was plotted in Fig. 8. It highlights the fact that IOUs have high values which are above 0.8. Additionally, 282 images out of 30,000 images were mis-detected during the detection stage. Furthermore, a distribution of confidence scores was plotted in Fig. 9. It is obvious that the detector has high confidence scores.

An object detection method is evaluated by calculating the detection precision and recall. In other words, the detector is considered optimal if it has high precision and high recall.

**Figure 10:**
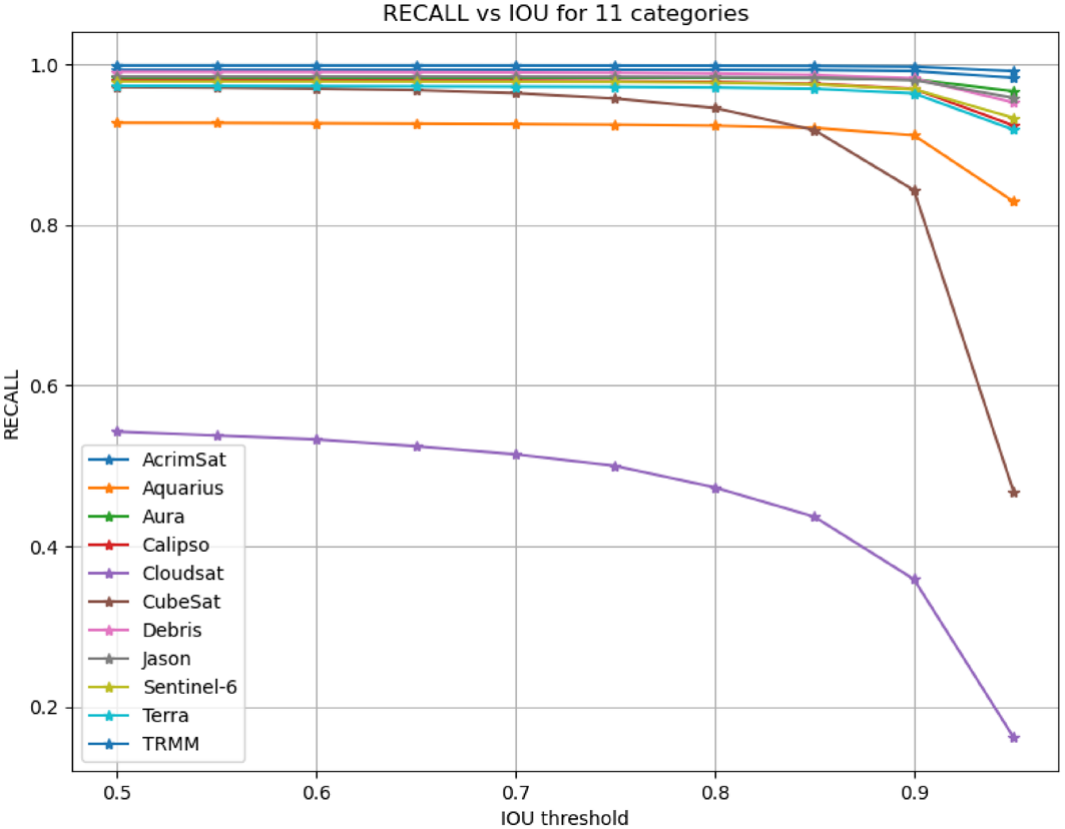
Recall vs. IOUs for 11 classes using the proposed solution.

The inability of a detection algorithm to detect objects present in the image by producing false negatives led to lower recall. In Table 6, the average recall of all classes at each IoU threshold was calculated and then the mean was determined. Additionally, we plotted recall against IoU on a curve with IoU thresholds on the x-axis and recall on the y-axis as shown in Fig. 10. This plot illustrates the recall for each class vs. IOU thresholds ∈ [0.5, 9.5].

**Table 6:** Recall and precision for various IOU values (0.5:0.95) using SPARK dataset.

IOU	Precision %	Recall %
0.5	93.89	93.88
0.55	93.26	93.82
0.6	92.33	93.76
0.65	90.95	93.65
0.7	88.92	93.51
0.75	85.96	93.29
0.8	81.17	92.89
0.85	73.55	92.20
0.9	59.00	90.46
0.95	25.50	82.62
Mean	78.45	92.00

**Figure 11:**
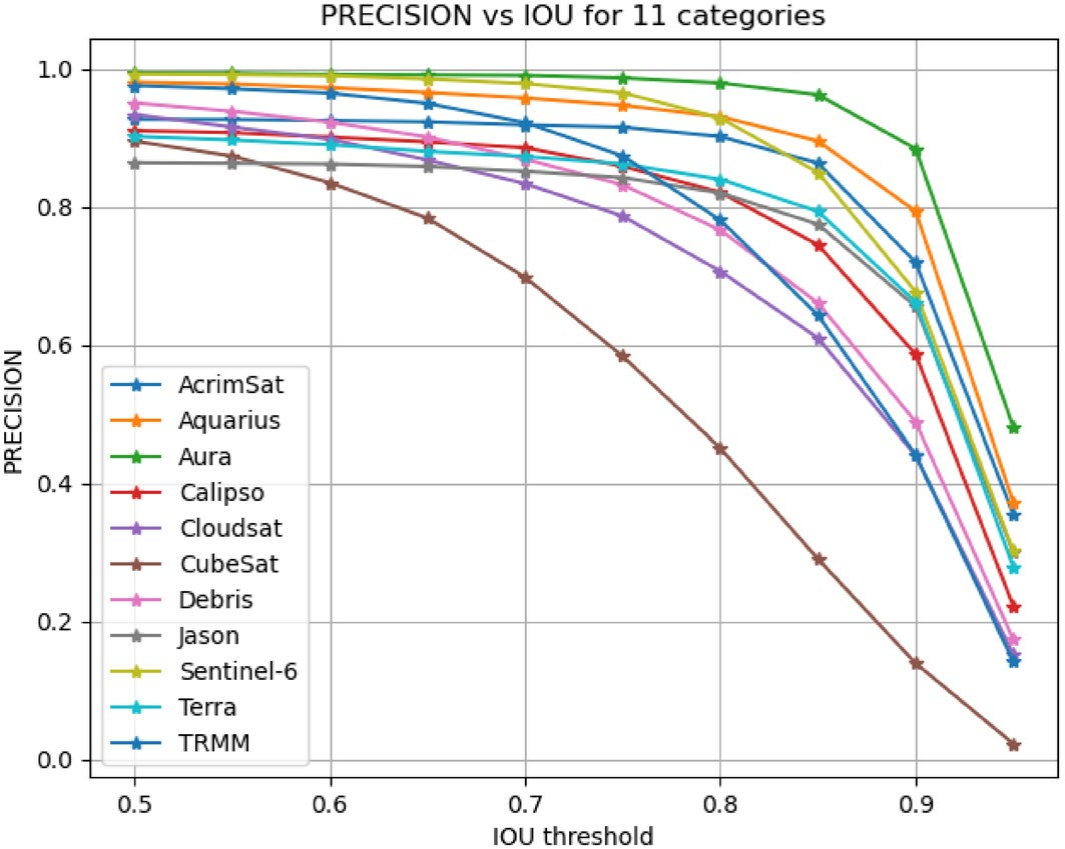
Precision vs IOUs for 11 classes using the proposed solution.

The wrong detection of irrelevant things in the background and labelling them with wrong object labels led to lower precision. Furthermore, smaller values of IoUs than the predefined threshold yield lower precision. In Table 6, the average precision of all classes at each IoU threshold was calculated and then the mean was found. Additionally, we plotted precision against IoU on a curve with IoU thresholds on the x-axis and precision on the y-axis as shown in Fig. 11. This plot illustrates the precision for each class vs. IOU thresholds ∈ [0.5, 9.5].

The limitation in EfficientDet with an EfficientNet-v2 backbone that was trained on the original training images was its inability to recognize images from the Cloudsat category well because of noisy and blurry images from this specific category in the testing set. Therefore, the Cloudsat category was misclassified and predicted wrongly with 107 correct predictions over 2500 images with an average accuracy of 89.5% for 11 categories. To address this problem, EfficientNet-B4 CNN was trained on cropped images after augmenting the images from the CloudSat category by blurring and adding Gaussian noise. As a result, the number of correct images from the CloudSat category was increased from 107 to 1334 images with an average accuracy of 91.54% for 11 categories.

Finally, the decision fusion approach was applied to combine decisions from both models—EfficientDet with an EfficientNet-v2 backbone and EfficientNet-B4 CNN. The final decision of final category was found by fusing two decisions and was found to increase the number of correct images from the CloudSat category to 1378 images with an average accuracy of 94% for 11 categories.

**Figure 12:**
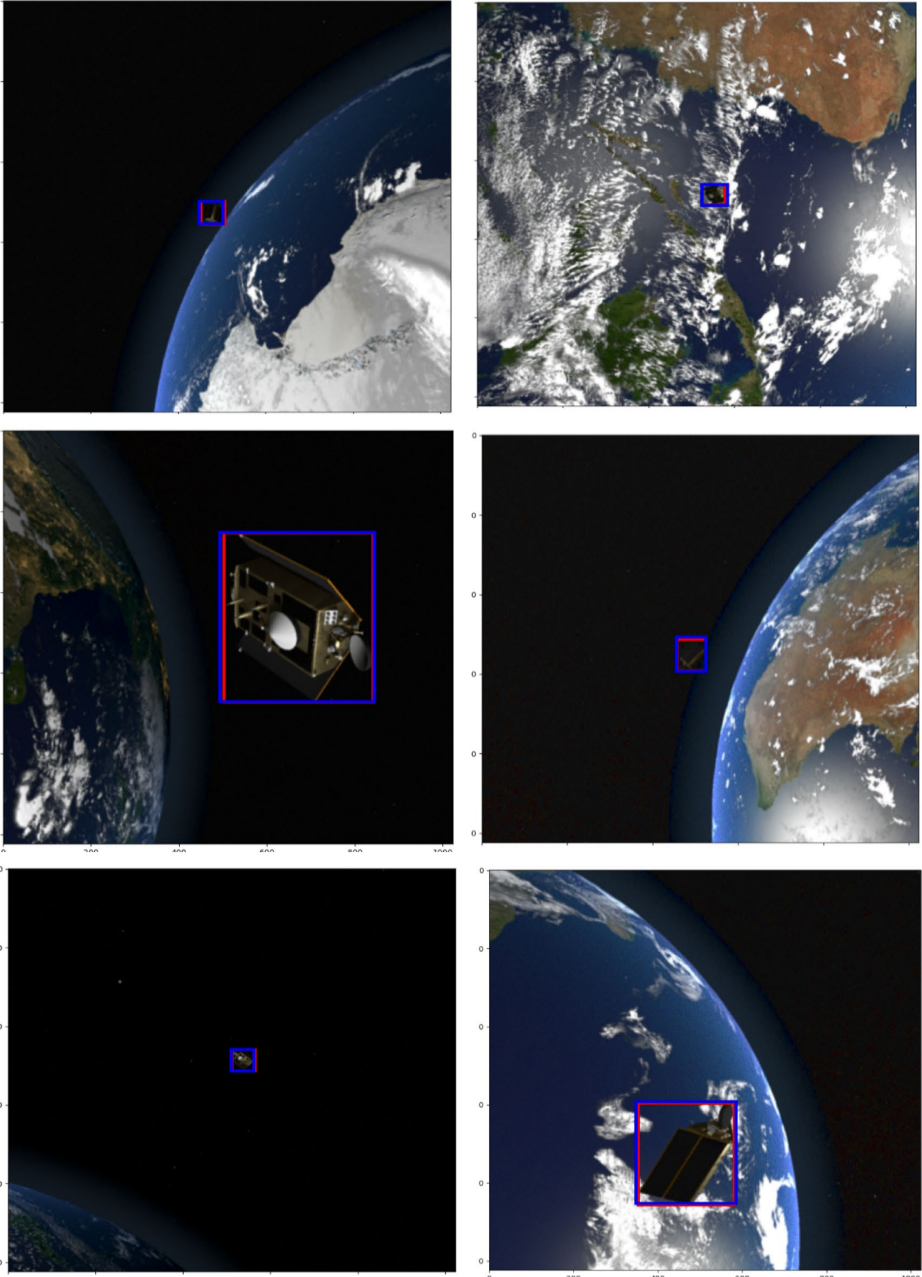
Few examples to show the overlap between actual bounding box (red) and predicted bounding box (blue) for the Sentine category^14,17,18^.

**Figure 13:**
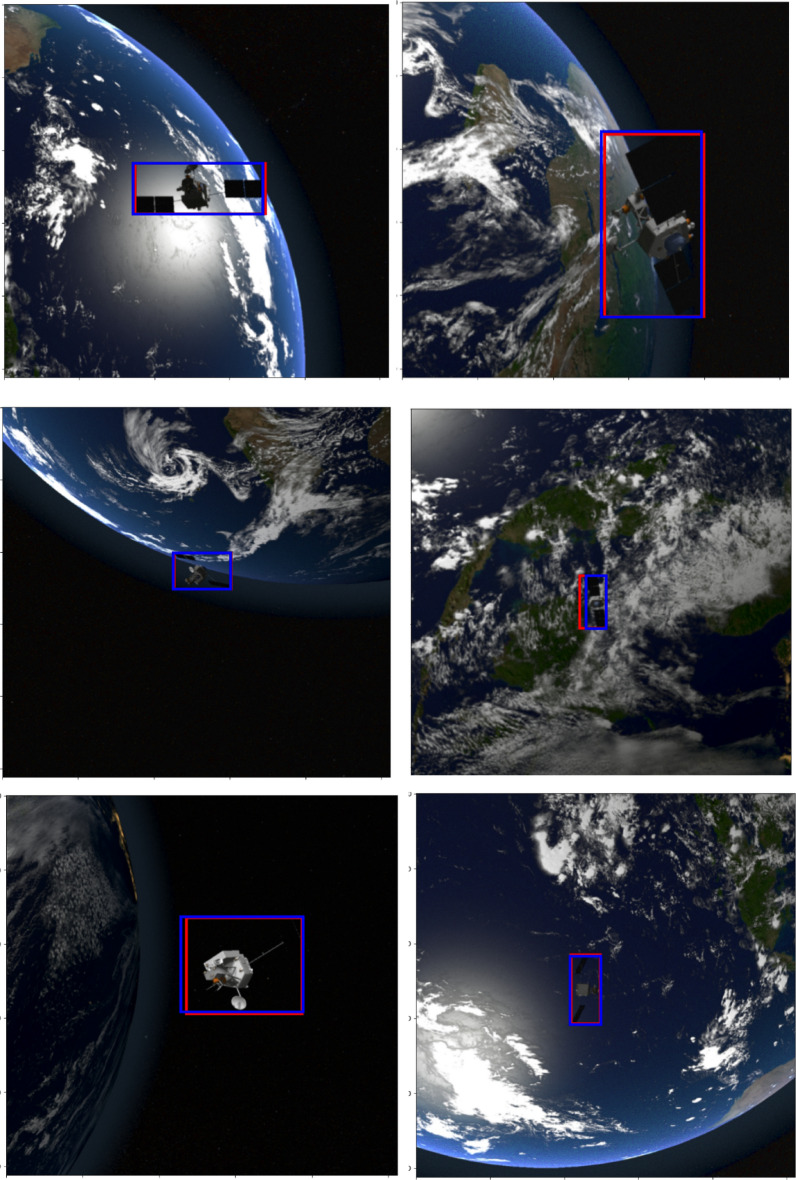
Few examples to show the overlap between actual bounding box (red) and predicted bounding box (blue) for the TRMM category^14,17,18^.

**Figure 14:**
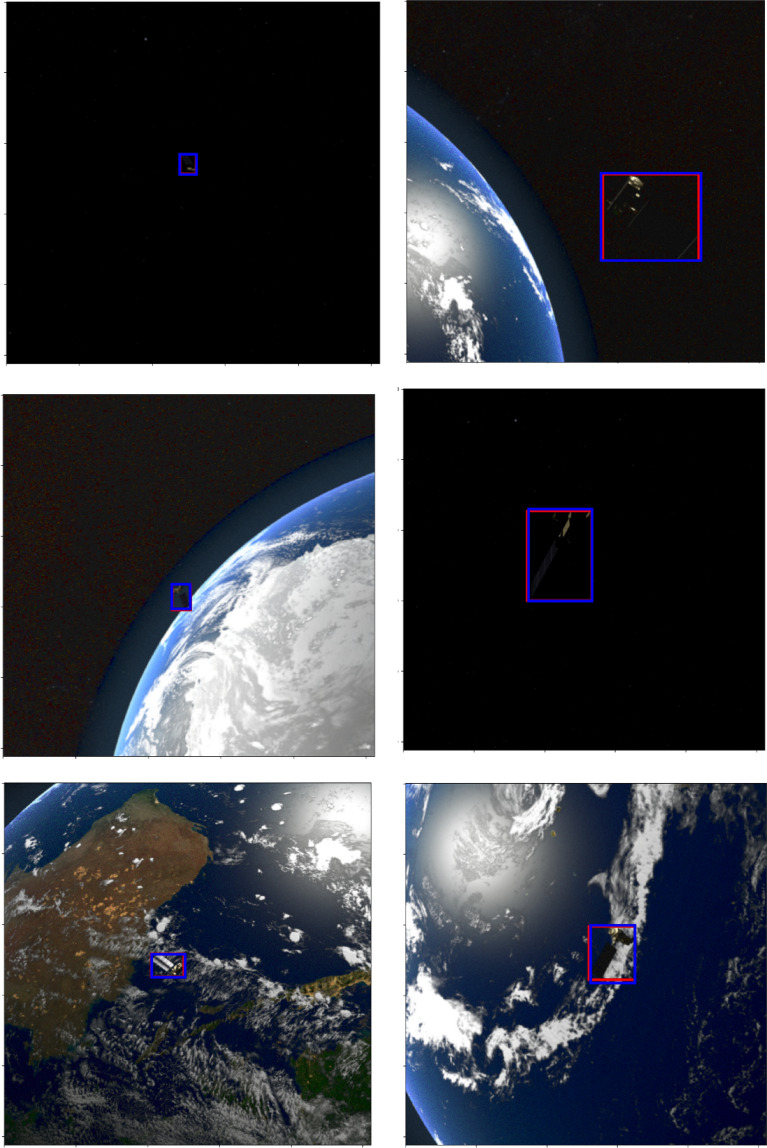
Few examples to show the overlap between actual bounding box (red) and predicted bounding box (blue) for the Terra category^14,17,18^.

Figures 12, 13, and 14 illustrate a few samples to show the overlap between actual bounding box (red) and predicted box (blue) for the Sentine, TRMM, and Terra categories. It is clear that EfficientDet was able to predict boxes that have large agreements with the ground truth boxes even if the backgrounds were complex as shown in the figures. Furthermore, the ability of the detector to localize small size objects belonging to various categories was behind the significant improvement in accuracy and performance metrics compared to existing methods. Even if several challenges were present in the dataset including random locations of objects, illuminated scenes and increased contrast, a variety of orbital settings, various positions and orientations of space objects in the background, and a substantial noise level, the detector was able to localize and classify space objects with favourable classification metrics: accuracy (94%), performance (1.9223); and detection metrics: mean precision (78.45%) and mean recall (92.00%).

The advantages of the proposed solution are that:

1. The task was formulated as an image detection problem. It can localize space objects by predicting the four coordinates of the box surrounding the spacecraft and debris. Additionally, it can classify the cropped images that contain space object into 11 categories. In other words, the proposed solution can focus attention on regions of interest (ROIs) that contain space objects inside the image and ignore irrelevant objects in the background. This matter plays a significant role in improving recognition accuracy.
2. The proposed solution can perform well in space missions because it is robust against all challenges present in this dataset including random locations of objects, illuminated stars and increased contrast, a variety of orbital settings, various positions and orientations of space objects in the background, the earth having oceans and clouds in the background, a substantial noise level, and various object sizes.
3. RGB images are enough to be used for the space object detection method. Therefore, there is no need for depth images that other methods utilized.

## Conclusion and future work

The study presented in this paper contributes to attract the research community by highlighting an interesting new challenge that enriches the body of knowledge. It proposed an efficient solution to localize and recognize space objects such as spacecraft and debris to enhance the performance of SSA system. In this research work, EfficientDet with an EfficientNet-v2 backbone was trained on the SPARK dataset to localize space objects in RGB images by predicting four coordinates of the boxes surrounding the objects. Additionally, a multi-modal learning approach is proposed for spacecraft classification using only RGB images to combine decisions from EfficientNet-v2 and EfficientNet-B4 that were trained on the SPARK dataset. The fused decision block was added to make the final decision about object class. We evaluated the proposed solution using various metrics for classification such as accuracy, and F1 score and for detection such as IOU, mean recall, and mean precision. Furthermore, we compared the proposed solution with other methods that utilized the same dataset.

An ablation study was done to validate the significant improvement in classification accuracy by using multi-modal learning which creates the final decision by combining decisions from efficientNet-v2 and EfficientNet-B4 CNNs. It was found that the proposed combination of EfficientDet with an EfficientNet-v2 backbone and EfficientNet-B4 CNN was able to outperform state of the art methods in terms of accuracy (94%), and performance metric (1.9223%) for object classification; and in terms of mean Precision (78.45%) and mean recall (92.00%%) for object detection. This study achieved its goal to enhance classification performance largely by focusing attention on regions of space objects and by ignoring other irrelevant objects in the backgrounds. Therefore, the proposed method of space object detector is a good feasible solution that can be utilized in real task of SSA system.

In the future, we plan to improve the performance of the solution by training recent object detectors such as YOLOv5 to evaluate its ability to detect space objects in this challenging dataset. Furthermore, to implement this solution on edge computing for real missions of SSA systems, we plan to train recent light versions of object detectors such as YOLOv5n^49^ and nanoDet^50^ using the SPARK dataset to balance between accuracy and inference speed.

## Data Availability

The Dataset belongs to University of Luxembourg and LMO. You may contact prof. Djamila Aouada (djamila.aouada@uni.lu) to request this dataset for research purposes.
